# Cd-Induced Apoptosis through the Mitochondrial Pathway in the Hepatopancreas of the Freshwater Crab *Sinopotamon henanense*

**DOI:** 10.1371/journal.pone.0068770

**Published:** 2013-07-22

**Authors:** Dongmei Liu, Jian Yang, Yingjun Li, Meng Zhang, Lan Wang

**Affiliations:** 1 Laboratory of the Bio-effect and Molecular Mechanism of Classical Environmental Pollutants, School of Life Science, Shanxi University, Taiyuan, Shanxi Province, China; 2 Institute of Molecular Biology, Nankai University, Tianjin, China; University of Jaén, Spain

## Abstract

Cd is one of the most common pollutants in the environment that also induces the apoptosis. To explore the mechanism of apoptosis in the hepatopancreas, freshwater crab *S* *. henanense* were treated with 0, 3.56, 7.12, 14.25, 28.49 and 56.98 mg/L Cd for 72 h. Apoptosis was noticeable in every treatment group and necrosis was observed clearly in the high concentration Cd groups. Classical apoptotic bodies were found by transmission electronic microscopy, which revealed chromatin condensation under nuclear membrane and mitochondrial membrane rupture. An increasing number of autolysosomes, damaged rough endoplamic reticulum and Golgi complex were observed as the Cd concentration increase. Brown colored apoptotic cells were detected by the TUNEL test in all Cd-treatment groups. The apoptosis index increased following the elevation of Cd concentration and got 32.9% in the highest Cd group. Caspase-9 and caspase-3 activities increased in the lower Cd treatment groups but no changes in the higher Cd concentration groups (comparing to the control group). The activity of caspase-8 did not change significantly. No significant change in the content of mitochondrial cytochrome c (cyt c) in Cd exposed groups except the decrease in the 56.98 mg/L group. In crabs treated with 3.56, 7.12 and 14.25 mg/L Cd, hyperpolarization of mitochondrial membrane potential (*Δψ*_*m*_) significantly increased. These results implied that apoptosis in the hepatopancreas induced by Cd occurrs through the mitochondrial caspase-dependent pathway. However, whether there are other apoptotic pathways needs to be studied further.

## Introduction

Cadmium (Cd) is a widespread environmental pollutant worldwide, which is discharged into the environment due to industrial, agricultural and urban activities. Since Cd is not degraded in the environment, it enters the body of animals and humans by respiration [1], occupational exposure and gastrointestinal absorption [2] and gets biomagnification through the food web [3]. Cd ions exert their toxic effects on tissues and cells where they are absorbed. Therefore, Cd is of increasing concern as an environmental toxicant. In aquatic ecosystems, several aquatic species show a huge bioaccumulation capacity of Cd [4,5]. High concentrations of Cd were reported in invertebrates such as oysters and other bivalve mollusks and crabs [6]. Sometimes, the content of Cd in crabs surpassed the maximum standard for Cd in crustaceans (Food Safety Law of the People’s Republic of China, GB1520l-94) [7]. The hepatopancreas of crabs was reported as one of the most important organs for Cd accumulation [4,8]. Cd intoxication in the hepatopancreas would endanger the life of crustacean because hepatopancreas is the main organ of detoxification and metabolism for crustacean.

The pathological, physiological, biochemical and genetic changes in different organs of freshwater crab, *Sinopotamonhenanense* , and the behavior of freshwater crab, *Potamonauteswarren* , were affected when exposed to Cd [9–13]. Cd-induced apoptosis was reported as well [14]. Apoptosis occurs through the extrinsic or death receptor-mediated pathway [15] and the intrinsic or mitochondrial-mediated pathway [16] is well-recognized. Other apoptotic pathways concerning endoplasmic reticulum [17], calcium [18] and ROS [19] were also reported.

Cd toxicity may induce mitochondrial damage and culminate in cell death either by apoptosis or necrosis [20,21]. Cd ions may induce the accumulation of reactive oxygen species (ROS) [22] which can lead to lipid peroxidation of membrane [23] and is harmful to the mitochondria [24]. Not only mitochondria are the site of energy production, but their roles in the regulation of apoptosis have been accepted [25]. Mitochondrial outer membrane permeabilization is considered an important initiative step in the apoptosis pathway [26]. Following the rupture of mitochondrial outer membranes, pro-apoptotic proteins are released from the mitochondrial intermembrane space, one of which was cytochrome c (cyt c), apoptosis-inducing factor (AIF) and second mitochondrial activator of caspases (Smac), etc. [27–29]. Subsequently, caspase-9, caspase-3 and poly (ADP-ribose) polymerase (PARP) could be activated and cleaved, which related to the mitochondrial pathway of apoptosis [30]. Translocation of AIF to the nucleus promotes DNA fragmentation independently of caspase activation [31]. Therefore, some researches suggested that apoptosis was triggered through the mitochondrial caspase-dependent pathway, and some were carried out through the caspase-independent pathway [27–29]. Excessive ROS production can also lead to mitochondrial membrane depolarization [24]. In several systems, apoptosis is associated with the loss of mitochondrial inner membrane potential (*Δψ*_*m*_), which may be regarded as a limiting factor in the apoptotic pathway. The vast majority of data were got from in vitro experiments. Even so, the precise role that Cd plays in these processes is only now starting to become apparent [20,32].

Crab is always regarded as a suitable bioindicator for aquatic heavy metals pollution [33,34]. As far as we know, the studies about the in vivo mechanism underlying Cd-induced apoptosis were rare. The present study would provide experimental evidence for interpreting the mechanisms of apoptosis in the freshwater crab *S* *. henanense* when exposed to Cd by several different approaches: TUNEL assay, transmission electronic microscopy, caspase-3, caspase-8 and caspase-9 activities, content of mitochondrial cyt c and mitochondrial membrane potential (*Δψ*_*m*_).

## Results

### Histopathological changes

**Figure 1:**
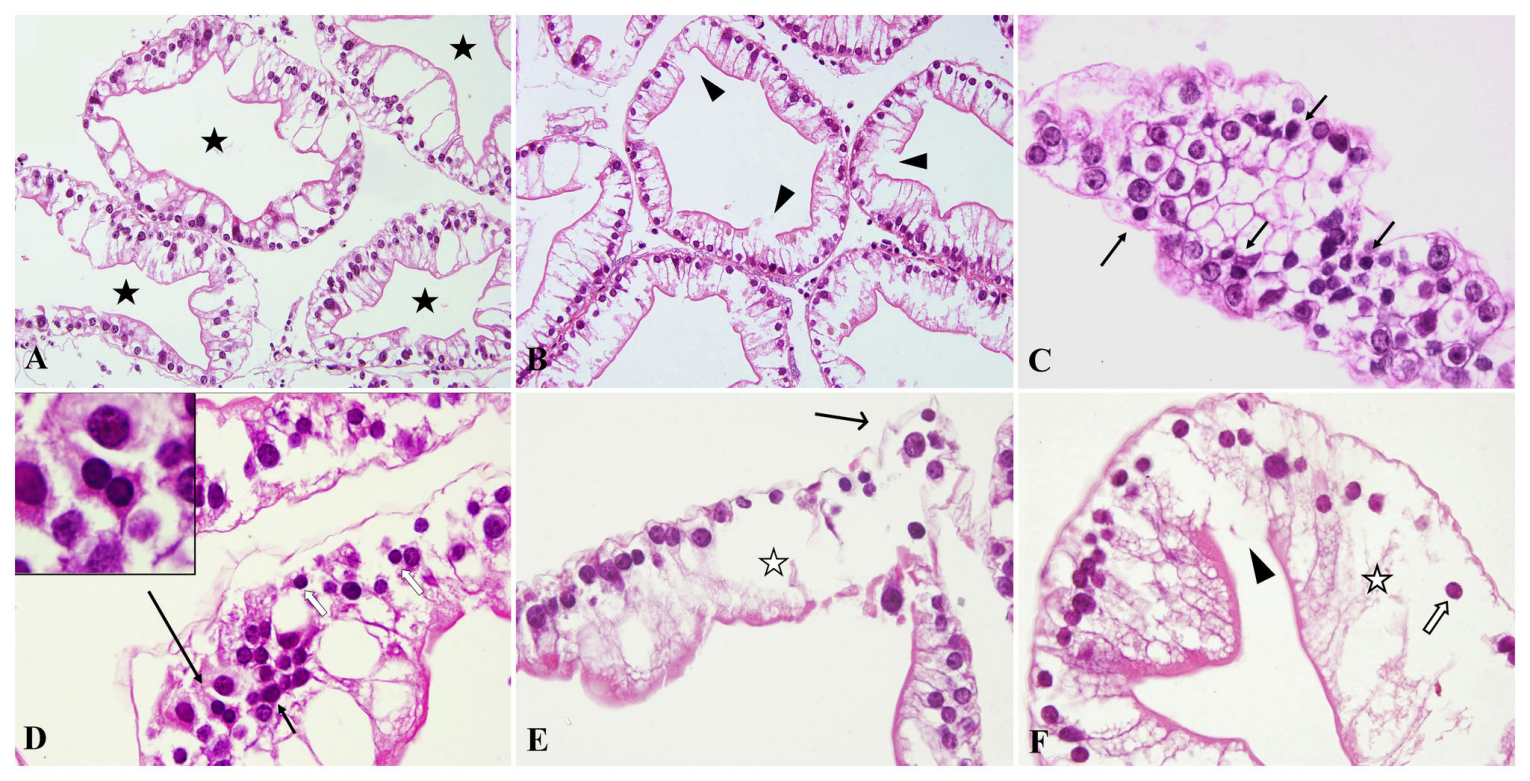
Morphological injury induced by Cd in *S* *. henanense* hepatopancreas with paraffin sections and HE staining (*n* = 6). (**A**) Normal tissue in the control group, ×200. The hepatopancreas glands were circular or elliptical, and the inner surface was irregular but continuous. (**B**) A few cells were damaged and the continuation of the inner surface was broken in the 3.56-mg/L Cd group, ×200. (**C**) Transverse section of the hepatopancreatic gland in the 3.56-mg/L Cd group, ×600. Cells arrange like the honeycomb. Classical apoptosis bodies (arrow) with chromatin aggregation and more acidophilia of cytoplasm were smaller than the surrounding ones. (**D**) Apoptosis was easily identifiable in the 7.12-mg/L Cd group, ×600. Classical apoptotic bodies are shown in the upper left corner. (**E**) Severely cellular swelling and damage were present in the 28.49-mg/L Cd group, ×400. (**F**) Broken epithelia, grave cell swelling and necroptosis were present in the 56.98-mg/L Cd group, ×600 (★: hepatopancreas glandular cavity; ◄: broken epithelia; ➞: apoptosis; ☆: severely cellular swelling; ➔: deciduous cells; ⇧: necroptosis).

The normal hepatopancreas glands shaped like a circle or an ellipse and the inner surface was irregular but continuous. Cells were lined up in order (Figure 1 A). In crabs exposed to the lower Cd concentrations, the epithelia were slightly damaged with dots of broken or swollen cells, maintaining, however, the tissue structure (Figure 1 B). Apoptosis, cellular swelling and necrosis apparently emerged as the Cd concentration increased. Apoptotic cells became smaller, contained more acidophilic cytoplasm and exhibited chromatin aggregation under karyotheca. Classical apoptosis was clearly identified in every treatment groups (Figure 1 C and D). The slightly edematous cells were bigger than the normal cells with the light staining of the cytoplasm but the almost normal nucleus. As the Cd concentration increased to 28.49 and 56.98 mg/L, the severely dropsical cells showing the bigger volume and transparent cytoplasm were called ballooning degeneration (Figure 1 E, F). Necrosis deteriorated from individual to focal of cells and the infiltration of the hemolymph cells could be observed in the interstitial tissue around necrosis in 28.49 and 56.98 mg/L groups. Concurrently, necroptosis with pycnotic nucleus and swelling cytoplasm was observed in some hepatopancreatic cells (Figure 1 D, F).

### Terminal deoxynucleotiodyl transferase dUTP nick end labeling (TUNEL) assays

**Figure 2:**
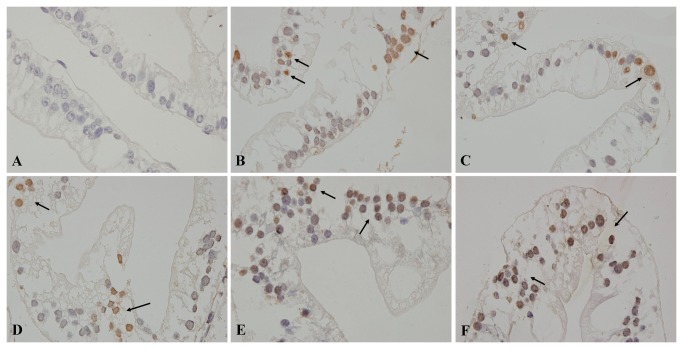
TUNEL assay for detection of the effect of Cd on hepatopancreas apoptosis in *S* *. henanense* (*n* = 4). (**A**) Control group. (**B**) 3.56-mg/L group. (**C**) 7.12-mg/L group. (**D**) 14.25-mg/L group. (**E**) 28.49-mg/L group. (**F**) 56.98-mg/L group. The apoptotic cellular nucleus was stained brown, ×600. The positive cells were dispersedly distributed or clustered.

Non-apoptotic nuclei were blue in hematoxylin counterstaining. There were few positive cells in the control group (Figure 2A). Positive nuclei were dyed brown by DAB staining (Figure 2B–F). As the Cd concentration increased, the percentage of positive cells in 3.56, 7.12 14.25, 28.49 and 56.98 mg/L Cd groups reached to 10.2 ± 4.5%, 14.4 ± 5.5%, 17.7 ± 5.6%, 23 ± 4.6%, and 32.9 ± 4.8%, respectively, and the apoptosis index (AI) increased significantly in each Cd group compared with the control group (0.9 ± 0.5%) (*p* < 0.01).

### Transmission electron microscopy (TEM) observation

**Figure 3:**
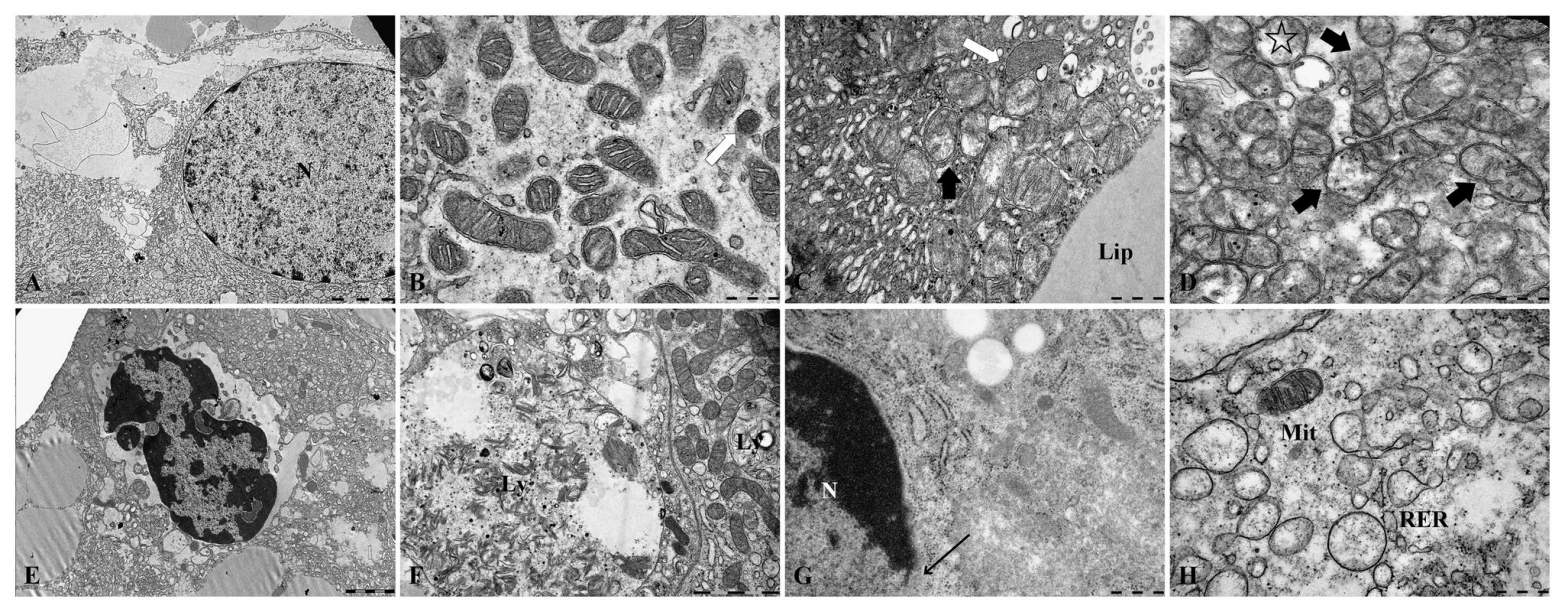
Pathological changes of the subcellular structure of the *S* *. henanense* hepatopancreas exposed to Cd. (**A**) Normal hepatopancreatic cell nucleus. Bar = 5 µm. (**B**) Normal mitochondria with a matrix of moderate electron density. Bar = 500 nm. (**C**) Mitochondria were damaged in the 7.12-mg/L Cd group. Mitochondria showed a matrix with low electron density and discontinuous membrane (➨). Primary lysosomes were seen (⇧). Bar = 500 nm. (**D**) Mitochondria were damaged in the 14.25-mg/L Cd group. The majority of mitochondria maintained their structure, but with a swollen matrix, reduction of the matrix density, shortening of the cristae, and rupture of a subset of the mitochondrial membrane (➨). Cristae disappeared and double-membrane vesicles were noted (✰) in severely damaged mitochondria. Bar = 500 nm. (**E**) Apoptotic cell in the 14.25-mg/L group. Chromatin was aggregated under the karyolemma and apoptotic bodies formed. Several morphologically normal mitochondria were seen. Almost all mitochondria turned into vesicles, and other organelles also disappeared. Bar = 2 µm. (**F**) Shows the autolysosomes and mitochondrion in two cells of the 14.25-mg/L Cd group. Bar = 2 µm. (**G**) Nucleus of a necrotic cell in the 28.49-mg/L group. The nuclear membrane was ruptured (**←**) and most organelles were disappeared. Bar = 500 nm. (**H**) Damaged RER and Mit in the 14.25-mg/L group. Bar = 500 nm (Ly: lysosome; Lip: lipid; Mit: mitochondria; N: nucleus; RER: rough endoplasmic reticulum).

As shown in Figure 3 A and B, normal cells had homogeneous chromatin in a round-shaped nucleus, ellipsoidal mitochondria with clearly discernible cristae, moderate electron density matrix and a modest number of lysosomes. In crabs exposed to 3.56 mg/L Cd for 72 h, no apparent changes were detected, although few mitochondria with outer membrane disintegration and decreased matrix electron density were observed. As Cd concentration increased, more damaged mitochondria appeared, which possessed the swollen matrix, unequal reduction of the material density, shortening of the cristae and rupture of subset of mitochondrial membrane until cristae disappeared. Finally, the severely impaired mitochondria formed the double membrane vesicles and disintegrated (Figure 3 C and D). Classical apoptotic bodies could be observed clearly, which had the characteristics of shrunk and deformed nucleus, aggregated and sprouted chromatin, and dropped nuclear fragmentation (Figure 3 E). Simultaneously, almost all organelles vanished and only several mitochondria were left in the thin cytoplasm. Many secondary lysosomes emerged with the increase of exposure concentrations (Figure 3 F). Apparent necrosis was observed in the 28.49 and 56.98 mg/L-Cd groups. Floccular chromatin was aggregated in the karyoplasm. Nuclear membrane was ruptured and the continuity of the membrane was broken (Figure 3 G). Injury of the Golgi complex and rough endoplasmic reticulum developed noticeably in crabs exposed to higher Cd concentrations (Figure 3H).

### Caspase-3, caspase-8 and caspase-9 activities

**Figure 4:**
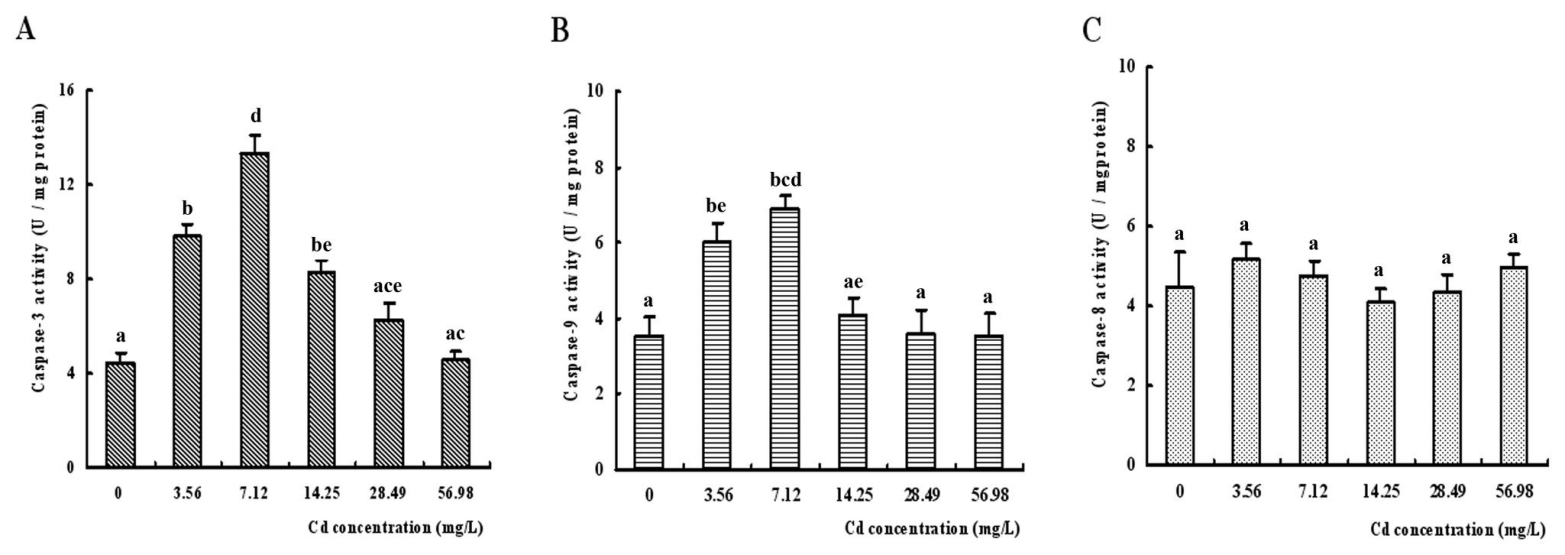
Effect of Cd on hepatopancreas caspase 3, 8, and 9 activities in *S* *. henanense* . (**A**) Caspase 3 activity (*n* = 6). (**B**) Caspase 9 activity. (**C**) Caspase 8 activity (The different small letters were used to indicate the significant difference (*p* < 0.05) between groups).

The activities of caspases-3, -8 and -9 in the hepatopancreas of *S* *. henanense* in different groups were shown in Figure 4. The activity of caspase-3 increased in the 3.56, 7.12 and 14.25 mg/L Cd groups and reached the maximum in the group 7.12 mg/L compared with the control crabs (*p* < 0.05). No significant difference was observed in 28.49 and 56.98 mg/L treated group (compared with control, *p* > 0.05). Moreover, the activity of caspase-3 decreased in 14.25 mg/L relative to 7.12 mg/L Cd group (*p* < 0.05) (Figure 4A). It was found that the caspase-9 activity in 3.56 mg/L and 7.12 mg/L Cd treatment groups rose compared with control group while the activities in groups 14.25, 28.49 and 56.98 mg/L Cd was significantly reduced relative to 7.12 mg/L group (*p* < 0.05) (Figure 4B). The activity of caspase-8 in Cd exposed groups did not change significantly compared with control group (Figure 4C). Significant positive relationship was detected between caspases-3 and -9 (r=0.945, *p* < 0.01), but no correlations between caspase-3 or caspase-9 and AI (r=0.037 and r=0.120, *p* > 0.05).

### Content of mitochondrial cyt c

**Figure 5:**
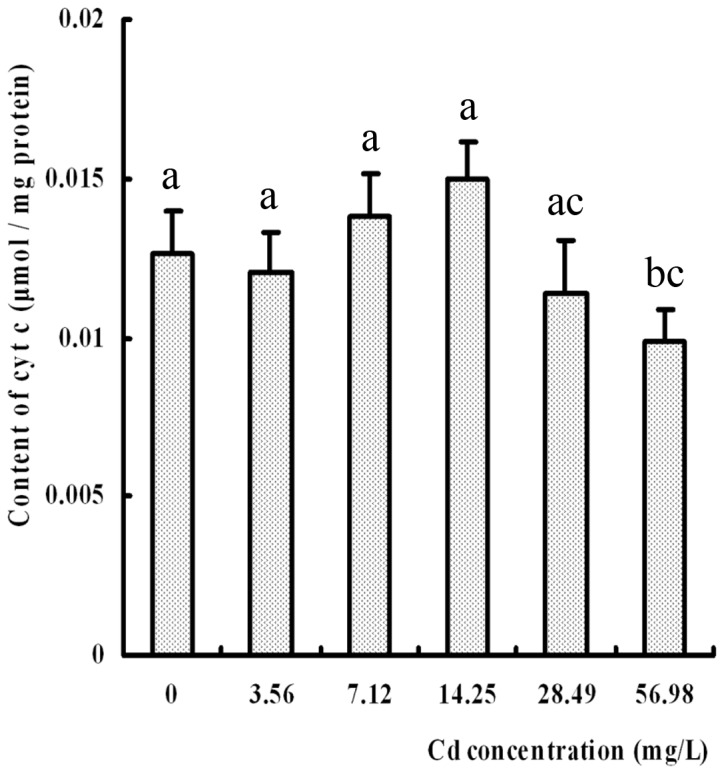
Effect of Cd on the content of cytochrome *c* in the *S* *. henanense* hepatopancreas. No significance between the Cd-treated and the control groups were observation except the 56.98 mg/L group markedly reduction, compared with control, different letter indicated *p* < 0.05 (*n* = 6).

Except for the group exposed to 56.98 mg/L Cd, where the mitochondrial content of cyt c decreased significantly when compared with the control group (*p* < 0.05), the alterations of the mitochondrial cyt c in other groups were not visible. But the content in the 14.25 mg/L group was slight higher than that of the control although there was no significant difference (*p* > 0.05) (Figure 5).

### Effect of Cd on *Δ*ψ_*m*_

**Figure 6:**
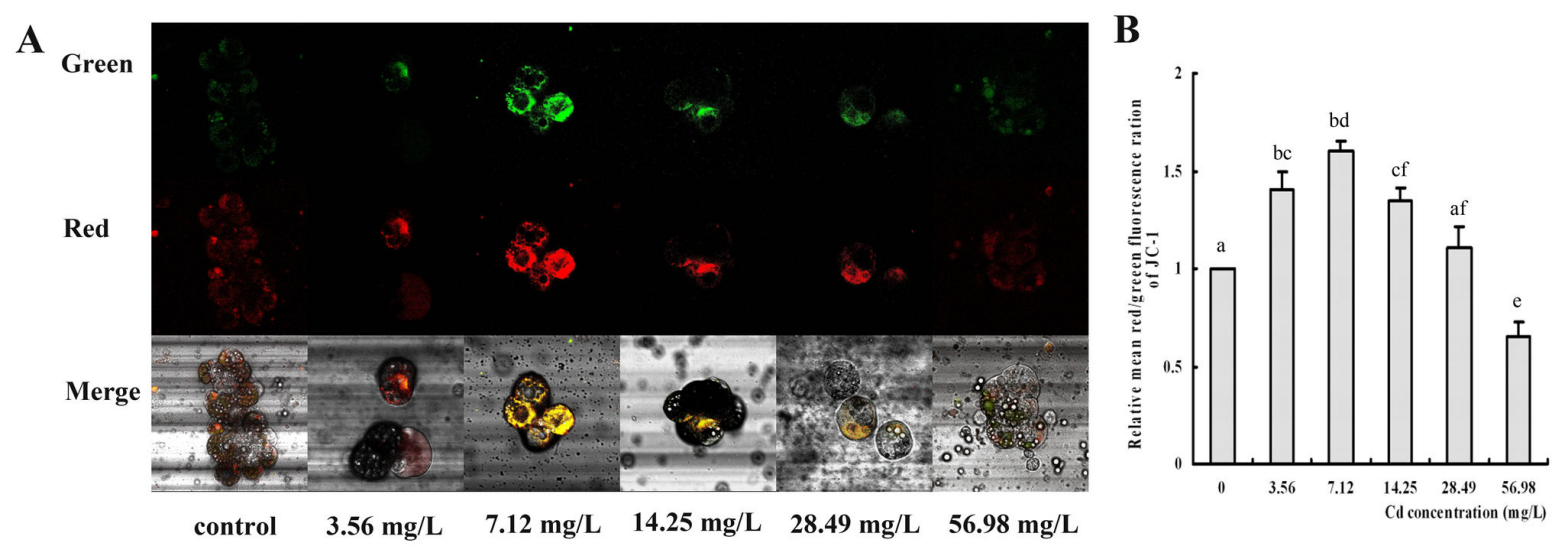
Mitochondrial membrane potential changes in *S* *. henanense* hepatopancreas cells labeled with JC-1 (*n* = 4). (**A**) Typical fluorescence micrographs captured by laser scanning confocal microscopy. Upper and middle panels show monomer (green) and aggregate (red) fluorescence, respectively. The other panels show the merged pictures of two images. (**B**) Histogram of the relative values of the mean red/green fluorescence ratios. The mean expression in each treated group is shown as fold increase compared with the mean expression in the control, which has been ascribed an arbitrary value of 1. Values are expressed as the mean ± SD. Significance was determined by one-way ANOVA (The different small letters were used to indicate the significant difference (*p* < 0.05) between groups, compared with control).

The changes of the fluorescence in the hepatopancreas of *S* *. henanense* are displayed in the Figure 6A. It can be seen in the merged images that the predominant fluorescence was red in the 3.56, 7.12 and 14.25 mg/L groups. Even the strong orange fluorescence was observed in the 14.25 mg/L Cd concentration, which suggested that *Δψ*_*m*_ was hyperpolarization. Then, the fluorescence intensity decreased and became weak in the higher concentration Cd groups, which indicated that *Δψ*_*m*_ decreased. The relative values of mean red/green fluorescence ratios were used to draw the histograms in Figure 6B. Compared with the control group, *Δψ*_*m*_ in 3.56, 7.12 and 14.25 mg/L Cd groups significantly increased by 1.40, 1.60 and 1.35 folds individually and decreased by 34.9% in the 56.98 mg/L Cd group (*p* < 0.05).

## Discussion

The freshwater crab *S* *. henanense* is widely distributed in the Yangtze River drainage, Huaihe River drainage and Yellow River Valley and lives in the sediments where it is easily exposed to pollutants such as the heavy metal Cd [35]. Cd caused several kinds of injury in the hepatopancreas of the freshwater crab *S* *. henanense* exposed to Cd for 72 h and the morphological changes had been described in detail in our other study [9]. Apoptosis, which occurs infrequently in the physiological state, was observed clearly in the hepatopancreas of Cd-treated crabs under the light microscopy. Moreover, hygropic degeneration and punctiform epithelium breakage were observed. The infiltration of hemolymph cells in the connective tissue in the higher Cd concentration groups indicated that necrosis and serious lesions occurred. Due to the limitations of the histological microexamination, the no-classical-change apoptosis in the early stage could not be recognized easily. A classical TUNEL test was used to determine apoptosis for the advantage of arresting DNA fragment early and sensitively [36]. The AI appeared to rise as the exposure concentration increased for the hepatopancreas tissue. However, the specificity of the TUNEL technique had also been criticized since it can produce a false-positive signal [37] because 3’-OH terminal on DNA chain can be stained in TUNEL assay as long as it was disclosed. In the groups of higher Cd concentration of this study, DNA breakage and 3’-OH terminal disclosed occurred accompanying with more serious impairments, such as cellular swelling, necroptosis and necrosis. These injuries may contribute to the increase of the apoptosis index in the higher Cd concentration groups [34]. TUNEL has been used successfully for detection of DNA degradation in paraffin-embedded tissue sections and can be combined with other methods if desired to allow more precise identification of apoptotic cells [38]. With the help of transmission electronic microscopy, apoptotic bodies and damaged mitochondria were observed in the treated crabs. The increase of mitochondrial outer membrane permeabilization is considered to be an initial step in the process of apoptosis [39]. Disruption of the outer mitochondrial membrane results in some apoptosis related protein redistributions from the mitochondria to the cytosol. Among these proteins, cyt c was a component of the mitochondrial respiratory chain, whose depletion committed the cell to a programmed cell death or a slower necrotic process due to a collapse of the electron transport system [40,41]. Another released protein, AIF, participated in caspase-independent apoptosis [42,43]. These two pathways ensure to execute the program of cell death effectively. We had no direct evidence to show whether other apoptosis related protein, such as AIF, p53 and JNK and MAPK etc., took part in the hepatopancreatic cells apoptosis, but apoptosis related to these proteins following Cd exposure were reported [28,31,44,45].

There are two distinct pathways for induced apoptosis: one starts with the ligation of death receptors leading to the activation of caspase-8 [46] and the other involves the mitochondrial release of cyt c enhancing caspase-9 activity [47]. Caspases-8 and -9 cleave and activate caspase-3 which kills the cells by cleaving a wide range of protein substrates [48–50]. Caspase-3, an effector caspase, has been shown to be critical in the course of apoptosis. Due to the activation of caspase-3 and the histological observation, it was concluded that Cd induced cell death via the apoptosis in crab hepatopancreas in the 3.56, 7.12 and 14.25 mg/L Cd groups. No changes in the activity of caspase-8 indicated that Cd-induced apoptosis in the crabs was not related to the death receptor pathway. Meanwhile, on account of the activation of caspase-9, apoptosis through mitochondrial pathway was definite in the crab hepatopancreas in the 3.56 and 7.12 mg/L Cd groups. Caspase-9 is an upstream activator and cleaves and cascades downstream effector, caspase-3 [51,52]. Therefore, caspase-3 could be cascaded by a little caspase-9 and the significant increased activity was measured although the significant change of caspase-9 was not detected in the 14.25 mg/L group. It was concluded that Cd-induced apoptosis occurred via mitochondrial caspase-dependent pathway in these three groups. In the groups exposed to 28.49 and 56.98 mg/L Cd, the results appear to conflict with each other, i.e., there were no activities of caspases-3 and -9 but morphological evidences of apoptosis. We presumed, therefore, Cd induced caspase-independent apoptosis or caspase-dependent apoptosis in the 28.49 and 56.98 mg/L Cd groups, where there were no caspases or the contents of caspases were not enough for detection. In addition, the conditions were more complex when considering the interference of the neural and humoral regulation in vivo. The conflicting alterations in the 28.49 and 56.98 mg/L Cd groups should be interpreted carefully and need to be studied further.

Cyt c participates in not only in the generation of energy but also in the induction of apoptosis. Cyt c is essential for caspase activation [53], which was decreased in mitochondria or increased in the cytoplasm of apoptosis [39,54]. The first report showing that cyt c plays a crucial role in the cell death pathway was published in 1996 [55]. Nevertheless, activation of caspase was not related to the level of cyt c in this study. It is well known that cyt c can be released and taken up by isolated mitochondria, while the reabsorption of cyt c restores mitochondrial function [56]. The released cyt c was quickly absorbed by normal mitochondria for the purpose of generating energy and increasing their resistance to stress. When animals are in stress, they acclimate themselves to survival at first. Various homeostasis and compensatory mechanisms were mobilized to prevent bodies from damages in the crab body. ATP is required for proteins biosynthesis, including metallothionein [57] and some enzymes, such as heat shock proteins, glutathione (GSH) synthesis [58], and possibly cyt c. Therefore, the decrease of cyt c in the mitochondria might be covered by reabsorption and/or synthesis in the 3.56, 7.12 and 14.25 mg/L Cd groups. If more mitochondria were damaged and more cyt c was lost, energy depletion and more serious cellular damage induced an obvious decrease of cyt c content in mitochondria of the highest concentration group.

*Δψ*_*m*_ is an important parameter for mitochondrial function and was measured by the optimal fluorescent dye, JC1-1 [59], in this study. Cd induced a significant increase of mitochondrial membrane hyperpolarization of crab hepatopancreas cells in the lower concentration groups. We assume that the increase of *Δψ*_*m*_ could represent a response to changes in energy demand after Cd exposure. Cannino et al. [60] got the similar results that Cd induced an increase of mitochondrial respiratory activity and mitochondria polarization in breast cells. In addition, some investigations also suggested that *Δψ*_*m*_ increases after induction of apoptosis, initiated by, e.g., calcium, Bax, ROS, and ceramides [61]. Apoptostic response to cellular stress is known to require ongoing protein synthesis and consumption of ATP [62]. Nargund et al. [63] also indicated that apoptotic response to Cd depended on continued metabolism of glucose. Cells could generate ATP quickly for the synthesis of more proteins [64] by digesting themselves. Cannino et al. [60] reported that Cd induced an increase of mitochondrial respiratory activity and mitochondria polarization in breast cells. We assume that the increase of *Δψ*_*m*_ could represent a response to changes in energy demand after Cd exposure. This also could explain why so many autolysosomes appeared in the hepatopancreatic cells. *Δψ*_*m*_ was reduced when there was more rupture of mitochondria at the higher Cd concentration. The results of our in vivo study were not absolutely the same as those of some previous in vitro studies.

## Materials and Methods

### Animals and treatments

Freshwater crabs *Sinopotamonhenanense* were purchased from Dong-an aquatic product market in Taiyuan City of Shanxi Province, China. In the course of 2-week acclimation, aerated water with pH 7.5 and dissolved oxygen 8.0-8.3 mg/L was exchanged every two days. Half of the aquarium was shielded using a black plastic to reduce visual disturbance. Crabs were fed every two days. Only healthy adult crabs with body mass 20.0 ± 0.5 g were sampled. Crabs were randomly divided into six experimental groups and allocated to control and 3.56, 7.12, 14.25, 28.49 and 56.98 mg/L Cd, corresponding to 1/32, 1/16, 1/8, 1/4, 1/2 of the 96 h LC_50_, respectively [39]. The exposure medium was not renewed and crabs were not fed during the period of the experiment. All other conditions were kept the same as those used for acclimation. Then four to six crabs were randomly sampled after 72 h of Cd-exposure from each group. Each assay was repeated three times.

After the 72 h exposure period, crabs were cryoanesthesized by putting them on ice for about 15 min. After opening the cephalothorax, the tissue samples of hepatopancreas were immediately excised for measurements or put into liquid nitrogen for later analysis.

### Histopathology

We processed the hepatopancreas for histological examination using standard protocols, fixed it in formalin, embedded in paraffin and sectioned the sample as 4 µm-thick slides using microtome (Leica RM2255, Germany). Five sections of each sample were deparaffinized in xylene, rehydrated in a graded alcohol series and stained with hematoxylin-eosin (HE). Digital images of ten non-overlapping visual fields were captured randomly from each section using light microscopy (Olympus BX 51, Japan) equipped the CCD (Olympus DP 71, Japan) and analyzed by an Image-Pro Plus 6.0 (IPP 6.0) analysis program (American, Media Cybernetics).

### TUNEL assays

Paraffin tissue sections, 5 µm in thickness, were mounted on slides coated with APES for TUNEL assay (Roche, Germany) by the kit guidance. After xylene dewaxing and gradient ethanol hydration, the sections were rinsed in distilled water for 5 min. The permeation of the slices in the citrate buffer (pH 6.0) was carried out in an autoclave for 3 min when the pneumatic mallet on the cover was elevated. In brief, sections were incubated in a TUNEL reaction mixture according to kit instructions and then treated with 3, 3-diaminobenzidine (DAB), and the reaction was observed under a microscope. The reaction was monitored and terminated in time, then the nuclei were counterstained with hematoxylin and the slices were dehydrated, transparent and mounted. Nuclei of apoptotic cells were stained by DAB and negative nuclei were counterstained with hematoxylin. Three sections were analyzed from each crab. Ten non-overlapping fields were selected randomly for each section and images were captured by a 60 X objective. Then, the apoptosis index (AI), percentage of the positive cells among all cells, was calculated in different Cd-treatment groups.

### TEM observation

Following dissection, 10 to 20 pieces of the tubules of the hepatopancreatic tissue were immediately fixed in 2% (v/v) glutaraldehyde for 2 h and postfixed in 1% (v/v) osmic acid. After dehydration in a graded series of acetone, samples were rinsed in propylene oxide and impregnated with epoxy resins. Two ultrathin sections, 50-60 nm, per crab were cut with an ultramicrotome (LK8, Sweden) and stained with uranyl acetate and lead citrate. Electron micrographs were taken with a JEM-1011 transmission electron microscope (Japan). About forty non-overlapping visual fields were captured randomly from each section.

### Caspases-3, -8 and -9 activities

Activities of caspases-3, -8 and -9 were measured by a corresponding Caspases Activity Assay Kits (Beyotime, China). The tissue samples of the hepatopancreas were collected, weighed and homogenized (5% w/v) in lysate, with a Potter-Elveh-jem type motor-driven homogenizer at 4 ^°^C. The homogenates were centrifuged at 15,000 g for 15 min at 4 ^°^C, and the supernatants were collected for caspase activity measurements. According to the description of the kits, one unit of enzymatic activity is the amount of enzyme that cleaves 1.0 nmol of the colorimetric substrate and produces 1.0 nmol *p*NA, which provides a characteristic absorption peak at 405 nm, per hour at 37 ^°^C and at saturated substrate concentrations. Optical density (OD) values were read by a multifunction microporous board detector (Spectra Max M5, America). Caspases activities were calculated using a standard curve. Protein contents were assayed according to the Bradford method [65].

### Measurement of content of mitochondrial cyt c

Appropriate amounts of hepatopancreas tissue was dissected and weighed immediately. Mitochondria were isolated from the hepatopancreas tissue by a Tissue Mitochondria Isolation Kit (Beyotime, China). Mitochondria isolation reagent (adding PMSF before use) was added into the tissue and the mixture was homogenized in a glass homogenizer. After homogenates were centrifuged at 1,000 g for 5 min, the supernatant was subsequently centrifuged at 3,500 g for 10 minutes yielding a precipitate of mitochondria. The mitochondrial resuspension with physiological saline was frozien and thawed 3 times to crush the mitochondria. The homogenates were centrifuged at 15,000 g for 15 min and the supernatants were collected and stored at -80 ^°^C in polypropylene tubes until assayed. The contents of cyt c in different Cd-treated groups were inspected by GENMED Cytochrome C Spectro-Quantification Kit (GenMed Scientifics, Shanghai Branch, China). There are two types of cyt c, i.e. oxidized and reduced. Reduced cyt c has the maximum absorption peak at 550 nm and is relatively stable. This characteristic is utilized to determine the content of cyt c. The calculation formula is provided by the kit. All the reagents were precooled and the operations were completed on ice or at 4 ^°^C. Protein contents were assayed according to the Bradford method [63].

### Measurement of *Δ*ψ_*m*_ by laser scanning confocal microscopy

About 50 mg of dissected hepatopancreas tissue was immediately put into the petri dish on ice containing an L-15 culture solution -20% bovine serum (L-15-BS). The glands were dilacerated along the long-axis with dissecting needles under an anatomical lens. The macroscopic tissue pieces were transferred into 500 µl L-15 -BS in polypropylene tubes and centrifuged at 1000 rcf for 3 min at 4 °C. The resuspended tissue pieces were incubated in 90 µl PBS with 10 µg/ml JC-1(final dilution) for 20 min in the dark. Centrifuged and cleaned three times in 1 ml PBS at 1000 rcf for 3 min, the tissue fragments were blended in 30 µl L-15 and reserved at 4 ^°^C for the assessment of the *Δψ*_*m*_ using a laser scanning confocal (Olympus FV1000, Japan) with a 40 X objective, zoom = 4 and 1024 X 1024 pixel resolution. 10 different microscopic fields were recorded and images were analyzed, measured and processed by the Olympus Fluoview Ver. 1.7 Viewer for semi-quantiﬁcation. Results were calculated according to the ration of red/green fluorescence.

### Statistical analysis

Statistical analyses were performed with SPSS15.0 software. Data were presented as mean values ± SD. Differences among groups were assessed by One-Way ANOVA followed by LSD. The correlations between caspase-3 and caspase-9, caspase-3 or caspase-9 and AI were analyzed by a Pearson test. Differences between means were regarded as significant if the *p* value was lower than 0.05.

## Conclusions

In summary, Cd induced mitochondrial caspase-dependent apoptosis in the hepatopancreas of the freshwater crab *S* *. henanense* . The apoptotic network is intertwined after 72 h Cd exposure. The critical point is likely around 14.25 mg/L Cd. Freshwater crab is tolerant of Cd and can be a sentinel species for heavy metal pollution monitoring. Whether there are any other pathways of apoptosis need to be studied further.
